# Principal component analysis of salivary cytokines and hormones in the acute stress response

**DOI:** 10.3389/fpsyt.2022.957545

**Published:** 2022-10-19

**Authors:** Rebecca Ryznar, Cheyenne Wong, Erin Onat, Francina Towne, Anthony LaPorta, Mark Payton

**Affiliations:** ^1^Department of Biomedical Sciences, Rocky Vista University College of Osteopathic Medicine, Parker, CO, United States; ^2^Rocky Vista University College of Osteopathic Medicine, Ivins, UT, United States; ^3^Rocky Vista University College of Osteopathic Medicine, Parker, CO, United States

**Keywords:** cytokines, chemokines, acute stress response, inflammation, principal component (factor) analysis

## Abstract

The acute stress response is characterized by activation of multiple interconnected systems in the body, resulting in the release of a flood of hormones and immune mediators into circulation. In addition to detection of these molecules in the serum, saliva can serve as a source of these markers as well and can be collected in a non-invasive way. The complete profile of salivary biomarkers associated with the hypothalamic pituitary adrenal/gonadal axes and the immune system during the acute stress response has not been fully elucidated. In a cohort of 62 first responders engaged in a stress training exercise, we set out to determine patterns of cytokine, chemokine and hormone shifts during the acute stress response. Salivary samples were collected immediately before (pre-stress), immediately after (post-stress) and 1 h after the stress test (recovery). Multiplex ELISA panels of 42 cytokines and 6 steroid and thyroid hormones were used to determine concentrations of these biomarkers during the three aforementioned time points. Principal components analysis was conducted to determine patterns in the large data sets collected. In our ≥0.3 loading principal components analysis, for pre-stress vs. post, post-stress vs. recovery and pre-stress vs. recovery, a total of three, four and three factors accounted for 56.6, 68.34, and 61.70% of the biomarker variation for each phase respectively. In the ≥0.7 loading principal components analysis, three, four and three factors were found for pre-stress vs. post, post-stress vs. recovery and pre-stress vs. recovery stages, respectively. Of note, in our ≥0.3 loading principal components analysis, MCP1 was present in all three factors from pre-stress to post-stress, and fractalkine was found to be in all four factors post-stress vs. recovery and pre vs. recovery from stress. Additionally, hormones testosterone, estradiol, T4 and T3 grouped together consistently in the same factor for all phases of acute stress in both ≥0.3 and ≥0.7 principal components analysis. Overall, our results identified specific patterns of immune markers and hormones that shift during acute stress and warrant further investigation to understand their mechanistic role in regulating the stress response.

## Introduction

Following a physical or psychological insult that disrupts homeostasis, the acute stress response is initiated. The acute stress response involves a coordinated series of psychoneuroimmunological events that provides organisms with a mechanism that results in physiological changes termed the “fight or flight” response. In particular, activation of the sympathetic-adreno-medullary (SAM) axis, the hypothalamus-pituitary-adrenal (HPA) axis, and the immune system will occur (1). Multiple studies suggest there is a bidirectional feedback loop between the HPA axis and the immune system (2–4). An acute mental or physical stressor that activates the HPA axis and ANS (autonomic nervous system) will induce an inflammatory response. Conversely, when the body is presented with infection, this primarily activates the immune system and will also induce neurobehavioral, neuroendocrine, and ANS responses. Cortisol, the primary stress hormone and glucocorticoid produced by the HPA axis, is necessary to suppress inflammation in response to stress. Studies have suggested neuroendocrine stress reactivity determines individualized immune signaling pathway variations (5).

In addition to changes in cortisol following acute stress, during acute stress (lasting minutes), certain kinds of cells are mobilized into the bloodstream, potentially preparing the body for injury or infection during “fight or flight” (1). Acute stress also increases blood levels of pro-inflammatory cytokines (6). Impaired immunity and higher levels of proinflammatory cytokines in circulation has been shown in individuals experiencing chronic stress and may lead to increased risk of infectious disease. Inflammation is a necessary short-term response for eliminating pathogens and initiating healing, but chronic, systemic inflammation represents dysregulation of the immune system and increases risk for chronic diseases and is associated with stress related-breakdowns (5).

The role of hormones other than cortisol, in the acute stress response, such as thyroid and gonadal hormones, is still not well understood. In addition, it is still not well known how these hormones affect the stress response through interactions with the immune system. The overall impact of stress on the thyroid occurs by slowing the body's metabolism, and thyroid functions slows down resulting in both triiodothyronine (T3) and thyroxine (T4) hormone levels falling (7). Additionally, many studies have shown that progesterone levels in the blood increased under stress conditions due to its secretion from the adrenal cortex (8). Sex hormones also reveal associations with the immune system and stress. Post-menopausal women have decreased expression of CD 4+ CD25+ T regulatory cells as a result of low estrogen levels. Studies done on males concluded that increased testosterone levels have been associated with an increased expression of CD 4+ CD25+ T regulatory cells (9). Decreased estrogen levels in younger women and post-menopausal women result in decreased expansion of regulatory cells. This makes them prone to autoimmune diseases (10). Stressful situations have been shown to cause a drop in testosterone levels, whereas the release of stress is shown to increase androgen levels (11, 12).

Measuring the acute stress response in humans is challenging, as access to the brain is both invasive and clinically non-viable. However, measuring salivary stress biomarkers provides an opportunity to capture stress response-related activity. Previous research has investigated single stress response biomarkers and linked salivary levels to individualized stress responses (13–15). While this remains important for translational research, single biomarker activity largely oversimplifies a complex pathophysiological process that has many individual components. A more comprehensive analysis of combined stress response biomarker activity has not yet been undertaken, and identifying techniques to capture the complete stress response is required.

One statistical approach that can be used to understand such complexity is principal components analysis. The primary purpose of principal components analysis is to define the underlying structure of data based on correlations between variables. In this context, it is a powerful tool to investigate interrelationships between salivary stress biomarkers without prior assumptions of likely associations. Principal components analysis can identify groupings of variables, which may help to identify specific patterns of factors or individual factors that may be critical in determining individualized responses to acute stress. Using this statistical technique on a cohort of first responders provides an in-depth analysis of the acute stress response in terms of biomarker activity. This analysis will elucidate how we might capture the complexity of acute stress response biomarker interrelationships and identify whether there are individualized responses. We believe that such an approach is required to investigate the key biomarkers involved in the acute stress response.

In this study, we aimed to use the principal components analysis statistical approach to characterize salivary stress response biomarkers in a cohort of first responders. We collected salivary samples immediately before stress (pre-stress), immediately after stress (post-stress) and 1-h post-stress stressful event (recovery). We employed principal components analysis to investigate the underlying structure and interrelationships of 42 cytokine and 6 hormone salivary biomarkers from these samples in order to better understand factors that may contribute to individualized acute stress responses.

## Materials and methods

### Subject population and study design

A total of 62 participants from a local fire department in Colorado, took part in the present study. All participants completed and signed consent forms to participate in the study; the study was reviewed and approved by the Rocky Vista University Institutional Review Board (IRB number: 2019-0092). Out of the 62 participants, 58 were male and 4 were female. Participants had a mean age of 30.6. The 77% of participants were Caucasian, 7% Black, 7% Hispanic, 2% Chinese, 6% Mixed race and 1% Indian heritage. Individuals in this study were fire academy recruits who participated in a stress-training test that required participants to pass in order to stay employed. Participants were blindfolded, distracted and had to navigate through a simulated collapsing home with a goal to keep their air mask on (this was deemed a pass for the test). This very high level of physical and emotional stress helps prepare fire recruits for training in actual burning buildings and for real life high stress scenarios on the job. This test is designed to push the fire academy recruits to the stress limit, while in a safe simulated environment. In an actual fire, the environment could be deadly if the participants removed their mask, as one or two breaths of super heated and poisonous smoke would render them unconscious. The study was performed over the course 1 year during 2020 in two separate cohorts, one in April 2020 and the other in June 2020. Three sets of salivary samples were collected. Samples were collected before the stressful event, directly after the event, and 1 h post-stress of the event. Inclusion criteria consisted of participants enrolled in the fire academy. All three salivary samples were collected in the morning, starting with the pre-stress samples, which were taken prior to the stress event, around 0800. The stress event took place within 2 h of the acquisition of the pre-stress samples, and immediately following its completion, the post-stress samples were taken. Lastly, the recovery samples were collected 1 h after the completion of the stress event.

Saliva was collected through the whole stimulated saliva method [reviewed in (16)]. For stimulated saliva sample collection, individuals were asked to chew sugar free gum for 5 mins, then 1 ml of saliva was collected and pipetted in 1.5 ml eppendorf tubes. After collection of the samples, a 1 μg/mL concentration of protease inhibitor was added and the samples were stored on ice (20–201 Millipore). The samples were shipped on dry ice and concentrations were determined using the bead based HD-42 cytokine plex panel and the HD-6 steroid/thyroid hormone plex assays from Eve Technologies (Alberta, Canada). Cytokines were reported in units of pg/μl, whereas hormones are reported in units of μg/μl. Sensitivities for each analyte are shown in Supplementary materials 1, 2.

### Statistical analysis

The data analysis for this paper was generated using PROC FACTOR in SAS Version 9.4 (SAS Institute, Cary, NC) for the following sample groups: pre-stress vs. post-stress (PreVPo), post-stress vs. recovery (PoVRe), and pre-stress vs. recovery (PreVRe). Descriptive statistics for each analyte and time point are shown in Supplementary material 3. The method for factor extraction was principal component analysis (PCA). Differences were calculated for each analyte and then those differences analyzed with PCA. Principal components analysis reduces large amounts of data into groups known as components or factors, based upon the amount of variation in the biomarkers that is attributable to each factor. By condensing the data into a smaller number of factors, it allows for analysis and interpretation of underlying patterns in the biomarkers. Principal component extraction was conducted with a default rotation approach (17). An eigenvalue indicates the amount of variance in the biomarkers explained by each factor; the factors are organized from highest to lowest eigenvalue. Thus, factor 1, which has the highest eigenvalue, explains the largest fraction of variance in the biomarker analysis for that sample group. The eigenvalues were utilized to calculate the proportion of variation explained by each factor (18). For an objective approach to determining the number of factors included in the graphical representations of the data, the factors explaining ≥10% of the biomarker variation as measured by the eigenvalues were included. Each biomarker is assessed by its' linear relationship within each factor, known as the “loading.” The loading of each biomarker within the factors represents the standardized regression coefficient when the factor is regressed on the biomarker (19). The initial interpretation of patterns in the data disregarded any loadings <0.3, which are considered weak to negligible. The preliminary investigation of the data was carried out with loadings ≥0.3, which are considered moderate loadings. Further analysis was carried out with loadings ≥0.7, which are considered strong loadings.

The biomarkers in each path diagram were analyzed for commonalities between the various factors including biomarker connections between factors, hormone grouping, and relative charges of the loadings. Analytes that were excluded from the analysis include: IL-17a and IL3 since all were OOR (out of range) below the minimum value determined by the standard curve. All other analyte values for each time-point were included in the analysis. The factor loadings for each analysis gives positive and negative loadings, which indicate whether the biomarker variation was in line with or in opposition to the pole of analysis respectively.

## Results

### Chemical fluctuations indicative of acute stress

**Figure 1:**
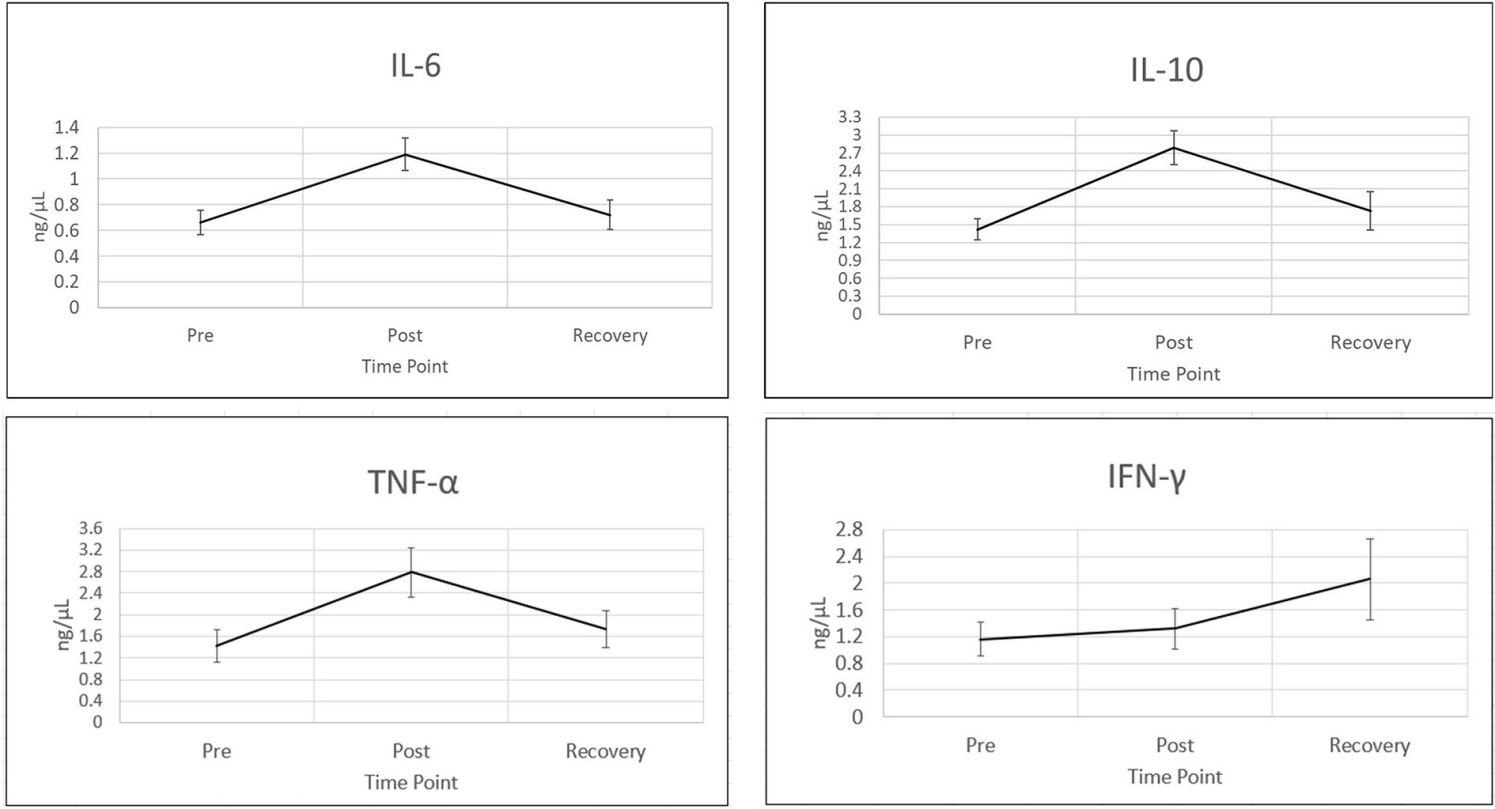
Cytokines increase during acute stress event. Four wellstudied cytokines known to be associated with acute stress (10), are shown in this figure. Time points are (1) pre-stress (immediately before stress test), (2) stress (immediately following stress test) and (3) recovery (1 h after stress test). All cytokines were measured using bead based multiplex Elisa from Eve technologies. Cytokine concentrations are in ng/μl. Representative error bars are +/– SEM (standard error of the mean). Sample sizes N for time point 1, IFNγ: *N* = 56, IL10, IL6 and TNFα: *N* = 57. All other analyte sample sizes and time points, *N* = 59.

In order to assess the effects of acute stress on salivary inflammatory markers, analyte concentrations from a multiplex panel of chemokines and cytokines were determined across three different time points during the event. Previous systematic reviews have shown that certain immune markers consistently increase during acute stress, including IL-6, IFNγ, IL-10 and TNFα (13–15, 20, 21). Mean concentrations for each of these analytes for pre-stress, post-stress and recovery time points are shown in Figure 1. For the four analytes displayed in Figure 1, all show an increase in salivary concentration from pre-stress to stress. IL-6, IFNγ, IL-10 and TNFα all increase during the stress time point and all but IFNγ decline almost back to baseline an hour after stress. IFNγ was the only cytokine that resulted in a dramatic increase even an hour after recovery. These results are consistent with a multitude of studies reporting specific cytokines that shift during the acute stress response (22). Biochemically, this stress training event supported the fact that participants were undergoing acute stress.

### Characterization of patterns in salivary biomarkers during the acute stress response with loadings ≥0.3 pre-stress vs. post-stress analysis

**Figure 2:**
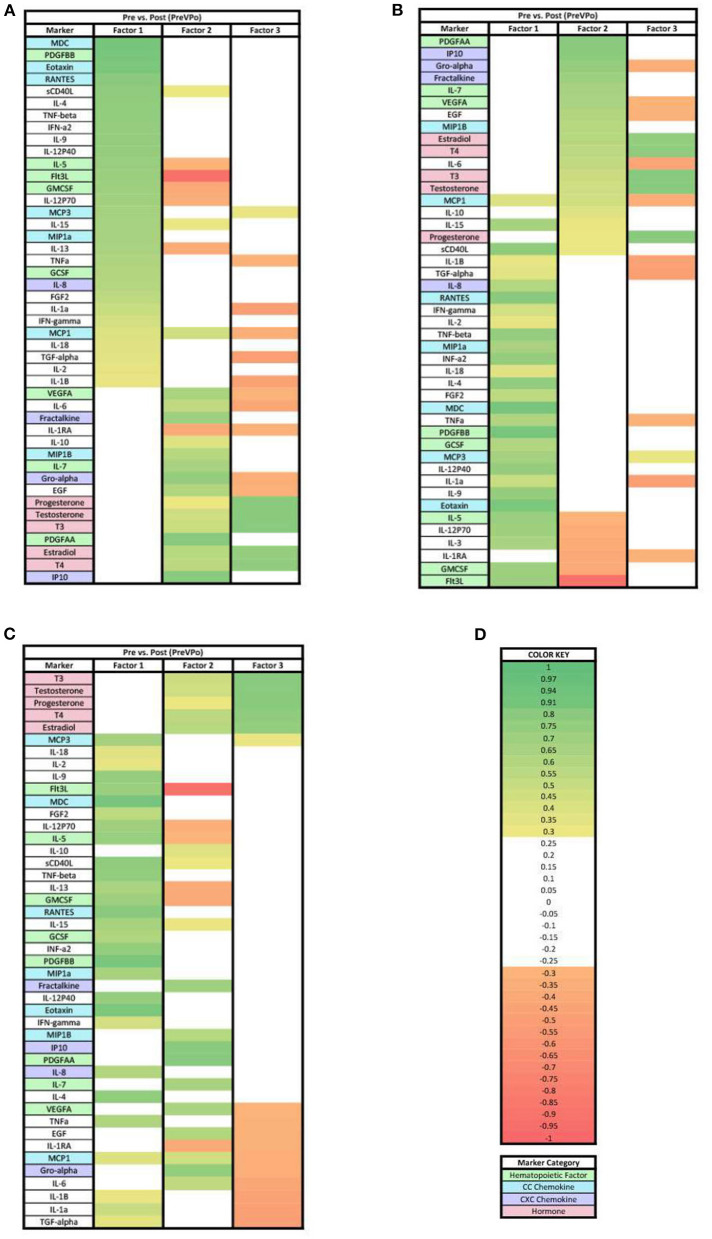
Heat maps of the pre-stress vs. post-stress (PreVPo) analysis of loadings ≥ 0.3. **(A)** Biomarkers sorted from highest to lowest loading in PreVPo factor 1. **(B)** Biomarkers sorted from highest to lowest loading in PreVPo factor 2. **(C)** Biomarkers sorted from highest to lowest loading in PreVPo factor 3. **(D)** Color key for PreVPo loadings ≥0.3, sorted top to bottom from most positive to most negative.

Our next step in the analysis was to combine all of the cytokine and hormone data into a larger data set for each time point, and perform principal components analysis. In the PreVPo analysis (Figure 2), three factors were found to account for 56.6% of the biomarker variation observed from the collected salivary samples (PreVPo factor 1, 28.28%; PreVPo factor 2, 15.97%; PreVPo factor 3, 12.39%). MCP1 was the only biomarker present in all three PreVPo pattern factors (Figure 2). All biomarkers in factor 1 were positive loadings (Figure 2A), while loadings in factors 2 and 3 were mixed positive and negative (Figures 2B,C). All tested hormones were positive and had loading factors ≥0.3 in both factor 2 and 3 for the PreVPo analysis (Figures 2B,C).

### Post-stress vs. recovery analysis

**Figure 3:**
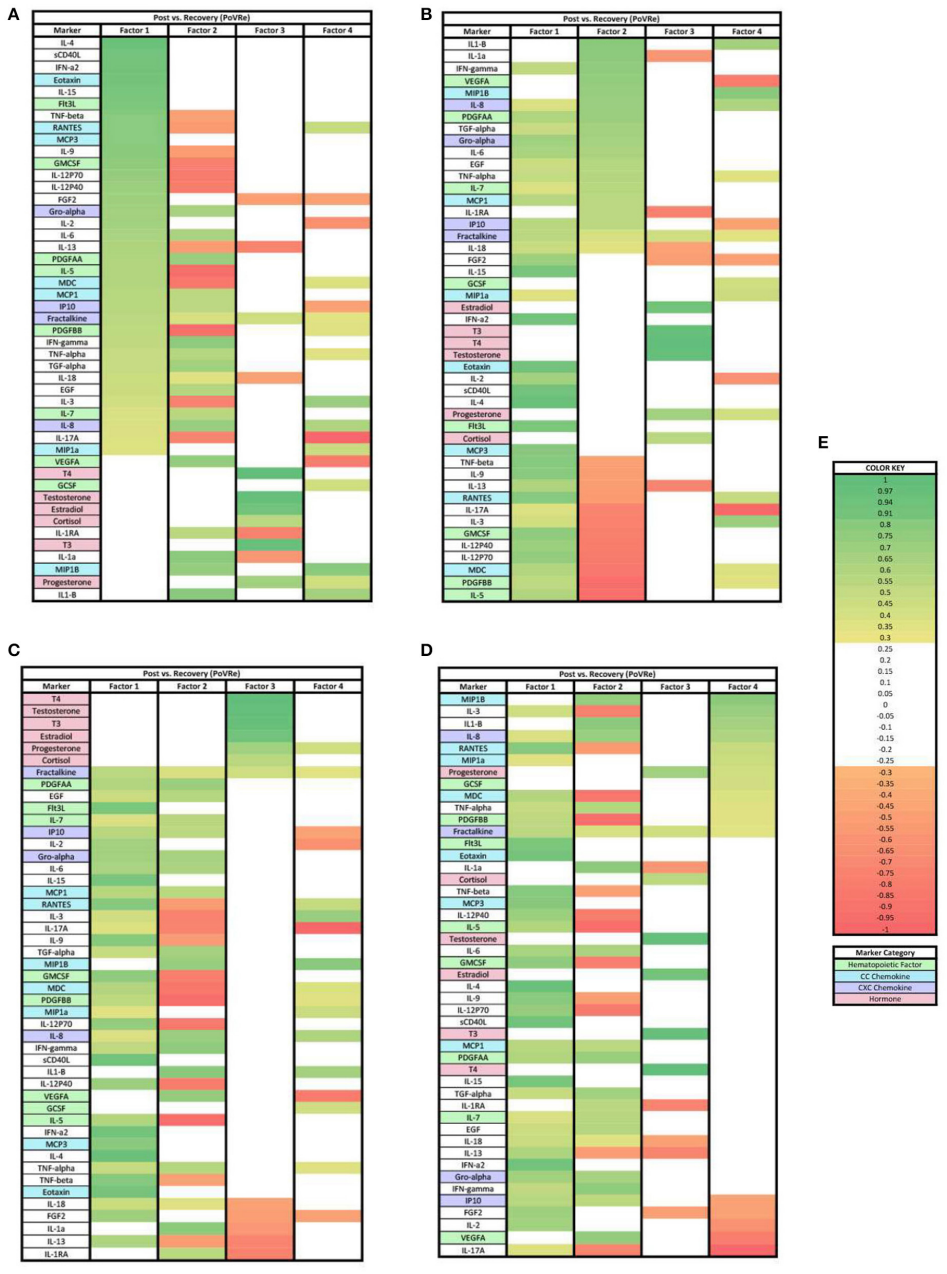
Heat maps of the post-stress vs. recovery (PoVRe) analysis of loadings ≥0.3. **(A)** Biomarkers sorted from highest to lowest loading in PoVRe factor 1. **(B)** Biomarkers sorted from highest to lowest loading in PoVRe factor 2. **(C)** Biomarkers sorted from highest to lowest loading in PoVRe factor 3. **(D)** Biomarkers sorted from highest to lowest loading in PoVRe factor 4. **(E)** Color key for PoVRe loadings ≥0.3, sorted top to bottom from most positive to most negative.

In the PoVRe analysis (Figure 3), four factors accounted for 68.34% of the biomarker variation (PoVRe factor 1, 26.70%; PoVRe factor 2, 19.74%; PoVRe factor 3, 11.82%; PoVRe factor 4, 10.08%). Fractalkine is present in all factors in the PoVRe analysis (Figure 3). All biomarkers in factor 1 were positive loadings (Figure 3A), while the loadings in factors 2–4 were mixed positive and negative values (Figures 3B–D). All hormones were positive and had loading factors ≥0.3 in factor 3 (Figure 3C), while the only hormone with a loading factor ≥0.3 in factor 4 was Progesterone, which was also positive (Figure 3D).

### Pre-stress vs. recovery analysis

**Figure 4:**
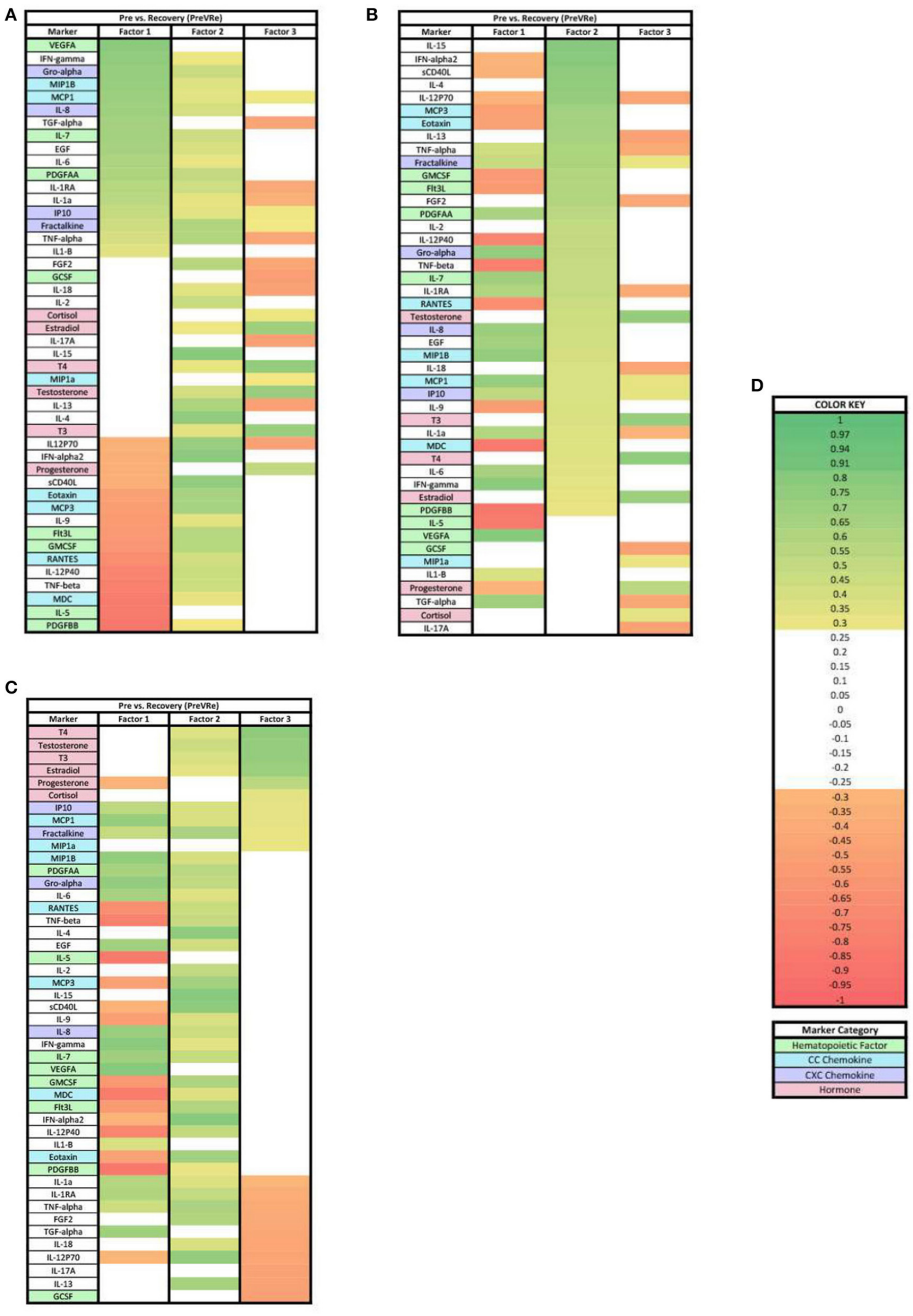
Heat maps of the post-stress vs. recovery (PreVRe) analysis of loadings ≥0.3. **(A)** Biomarkers sorted from highest to lowest loading in PreVRe factor 1. **(B)** Biomarkers sorted from highest to lowest loading in PreVRe factor 2. **(C)** Biomarkers sorted from highest to lowest loading in PreVRe factor 3. **(D)** Color key for PreVRe loadings ≥0.3, sorted top to bottom from most positive to most negative.

In the PreVRe analysis (Figure 4), three factors accounted for 61.70% of the biomarker variation (PreVRe factor 1, 27.19%; PreVRe factor 2, 23.19%; PreVRe factor 3, 11.33%). Fractalkine is present in all factors in the PreVRe analysis (Figure 4). All biomarkers in Factor 2 were positive loadings (Figure 4B) while the loadings in Factor 1 and 3 were mixed positive and negative loadings (Figures 4A,C). The hormones with loadings ≥0.3 were positive in factor 2 and 3 (Figures 4B,C), with factor 3 containing all tested hormones and factor 2 only containing Estradiol, T3, T4, and Testosterone. However, progesterone was a negative loading in factor 1 (Figure 4A).

### Characterization of patterns in salivary biomarkers during the acute stress response with loadings ≥0.7

**Figure 5:**
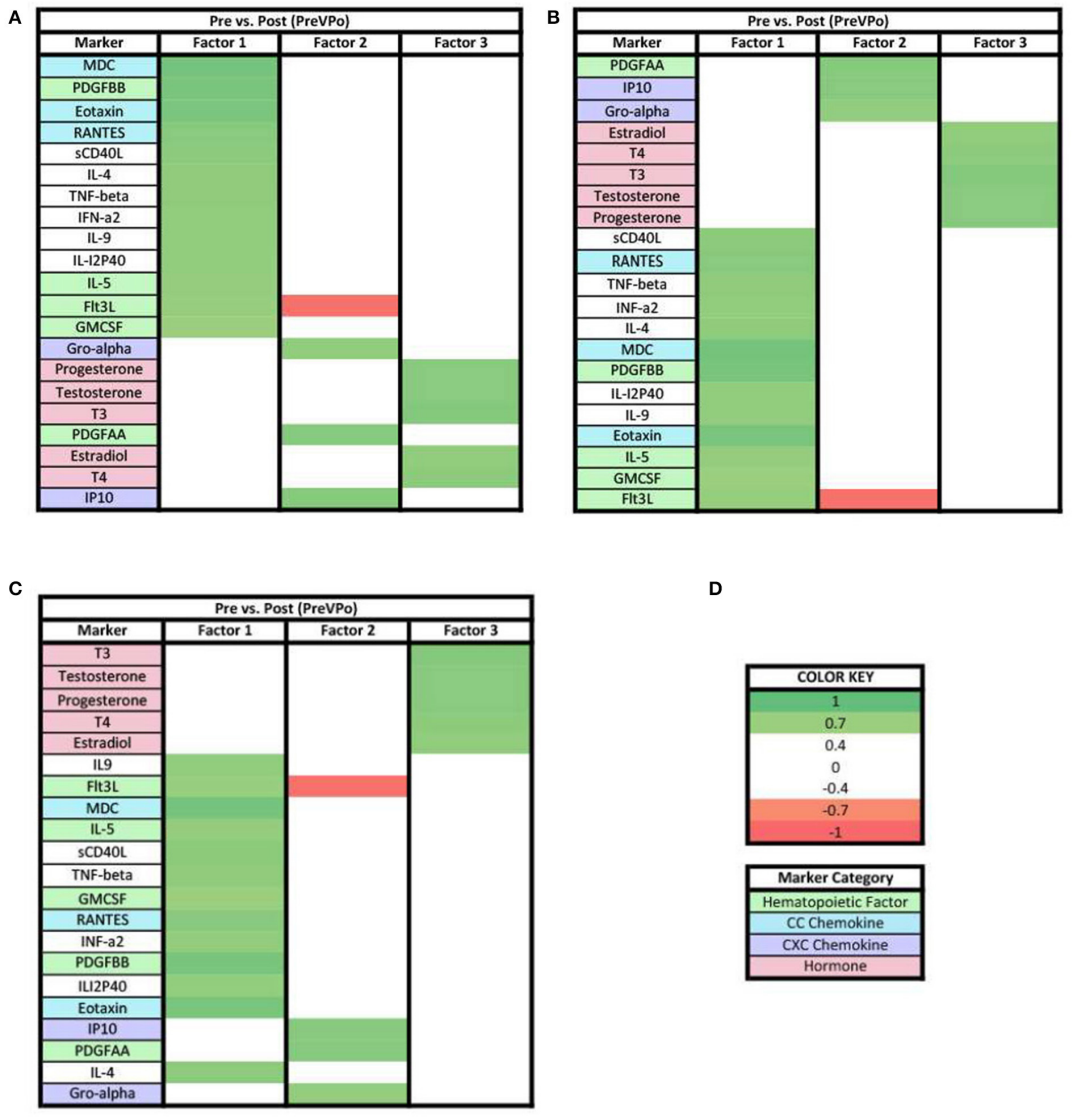
Heat maps of the pre-stress vs. post-stress (PreVPo) analysis of loadings ≥0.7. **(A)** Biomarkers sorted from highest to lowest loading in PreVPo factor 1. **(B)** Biomarkers sorted from highest to lowest loading in PreVPo factor 2. **(C)** Biomarkers sorted from highest to lowest loading in PreVPo factor 3. **(D)** Color key for PreVPo loadings ≥0.7, sorted top to bottom from most positive to most negative.

When evaluating the charges of the PreVPo analysis for the loadings ≥0.7, all three factors display positive loadings (Figure 5). The hormones with loading factors ≥0.7 were found together, with a positive charge in factor 3 (Figure 5C).

**Figure 6:**
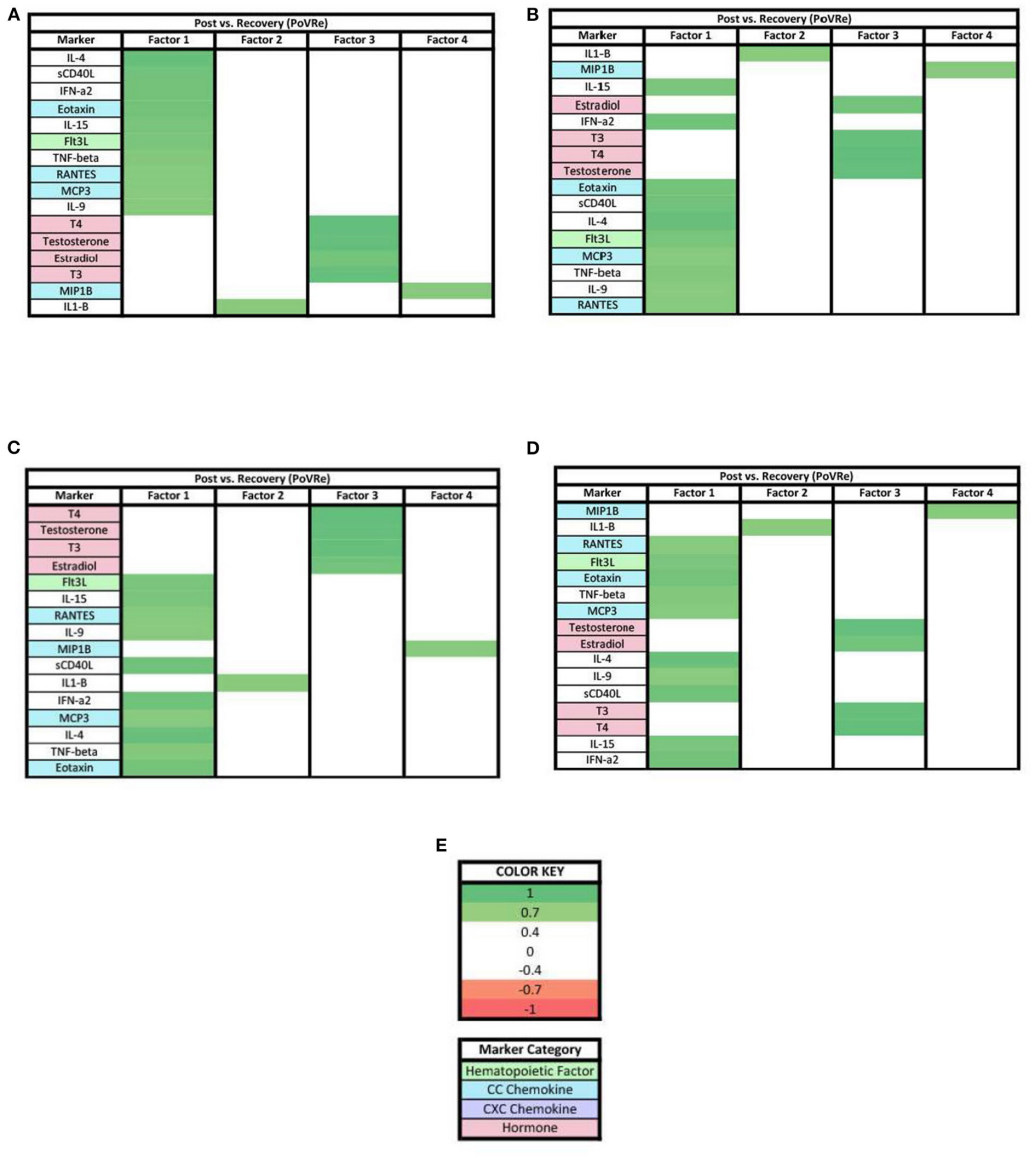
Heat maps of the post-stress vs. recovery (PoVRe) analysis of loadings ≥0.7. **(A)** Biomarkers sorted from highest to lowest loading in PoVRe factor 1. **(B)** Biomarkers sorted from highest to lowest loading in PoVRe factor 2. **(C)** Biomarkers sorted from highest to lowest loading in PoVRe factor 3. **(D)** Biomarkers sorted from highest to lowest loading in PoVRe factor 4. **(E)** Color key for PoVRe loadings ≥0.7, sorted top to bottom from most positive to most negative.

In the PoVRe analysis all remaining biomarkers with loadings ≥0.7 were positive loadings (Figure 6). In factor 3, Estradiol, T3, T4, and Testosterone had positive loadings ≥0.7 (Figure 6C).

**Figure 7:**
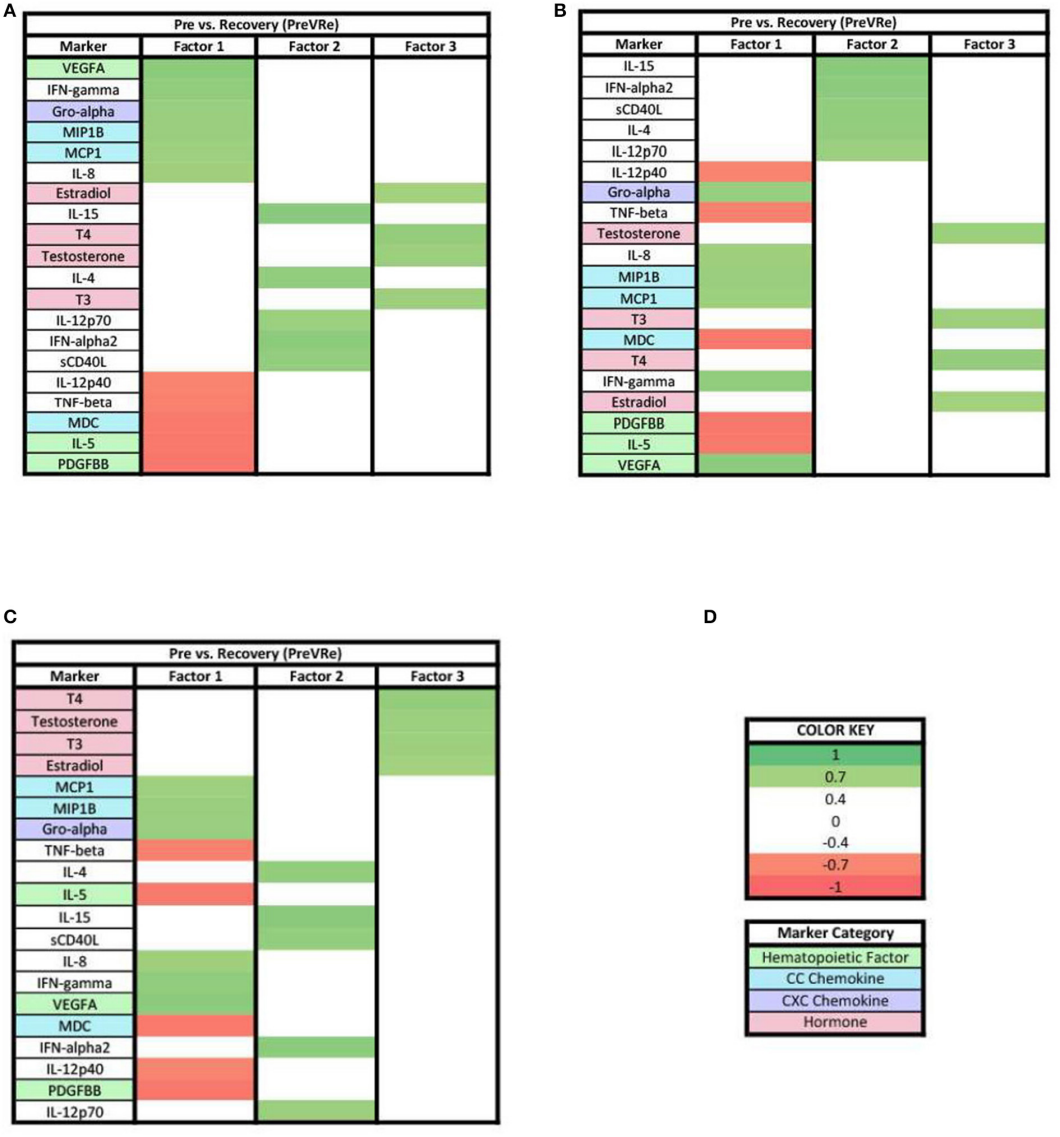
Heat maps of the post-stress vs. recovery (PreVRe) analysis of loadings ≥0.7. **(A)** Biomarkers sorted from highest to lowest loading in PreVRe factor 1. **(B)** Biomarkers sorted from highest to lowest loading in PreVRe factor 2. **(C)** Biomarkers sorted from highest to lowest loading in PreVRe factor 3. **(D)** Color key for PreVRe loadings ≥0.7, sorted top to bottom from most positive to most negative.

For the PreVRe analysis (Figure 7), the biomarkers in factor 1 were mixed with positive and negative loadings (Figure 7A), while all biomarkers in factors 2 and 3 were positive loading with the hormones Estradiol, T3, T4, and Testosterone having loadings ≥0.7 in factor 3 (Figures 7B,C).

### Changes from ≥0.3 to ≥0.7 loading patterns

When evaluating the patterns in biomarker factors within the analyses for biomarkers with loadings ≥0.7, the connection of MCP1 in the three PreVPo factors was no longer present (Figure 5). In fact, MCP1 was no longer found in any of the factor patterns except for factor 2 of the PreVRe analysis (Figure 7B). In addition, the connection of Fractalkine between all of the factors in the PoVRe and PreVRe analyses was no longer present (Figures 6, 7). Fractalkine was only found in factor 1 of the PreVRe analysis (Figure 7A). The ≥0.7 results revealed T3, Estradiol, and Testosterone in factor 2 of the PreVPo and factor 3 of the PreVRe analyses (Figures 5B, 7C). In addition, the PreVRe analysis showed those three hormones along with T4 in factor 3 of the PreVRe analysis (Figure 7C).

## Discussion

The purpose of this analysis was to examine the patterns of salivary immune markers and steroid/thyroid hormones during the acute stress response. In particular, we set out to understand how these markers shift during acute stress. In our study population, multiple factors were uncovered for each stage of the acute stress response. We investigated a combination of both immune markers and hormones produced through the HPA/HPG axes, since a bidirectional feedback loop exists between these two systems to regulate the response to stress. Interpretations for each phase of the acute stress response are discussed below.

### Factors for pre-stress vs. post-stress in acute stress response

In both ≥0.3 and ≥0.7 loading analyses, three factors were found in the PreVPo analysis (Figures 2, 5). For the ≥0.3 analysis (Figure 2), factor 1 was made up of a grouping of proinflammatory cytokines that included primarily CC chemokines (MDC, Eotaxin, RANTES, MCP3, MIP1a), cytokines (IL-4, IL-9, IL-18, etc) and one strong CXC chemokine (IL-8) as well as markers associated with central hematopoiesis [Flt3, IL-5, GMCSF, IL-7 (23)]. In factor 2, there is pattern of pro-inflammatory cytokines including some CXC chemokines (IP10, Gro-alpha, fractalkine) with an addition of some anti-inflammatory markers (IL-10), steroid and thyroid hormones, along with peripheral hematopoietic markers as well (PDGFAA, VEGF). Factor 3 showed a decrease in pro-inflammatory markers, along with a very tight grouping of the hormones.

Of note for the ≥0.3 loading analysis, we found that the change in MCP1 from pre-stress to post-stress was correlated with all 3 factors but one of these loadings is negative, suggesting that MCP1 changes are positively correlated with changes in analytes loading positively to factors 1 and 2, and negatively correlated with changes in other analytes that have positive loadings for factor 2. MCP1 is seen in all three factors for this specific phase of the acute stress response (Figure 2). MCP1 or monocyte chemoattractant protein 1, is a known proinflammatory chemokine that recruits leukocytes including monocytes, dendritic cells and memory T cells to sites of inflammation. More specifically, MCP1 may play a role in the ability of astrocytes to recruit peripheral monocytes during the stress response (24). In a recent published review, MCP1 was mentioned as a marker associated with low resilience to stress, specifically, the salivary level of MCP1 was found to be correlated with PTSD associated symptoms in multiple studies (24). Lower levels of MCP1 have been found to be associated with greater happiness during acute stress (25). Overall, our PreVPo results suggest immediately before stress to peak of acute stress, hematopoiesis and CC chemokines are most important for regulating the response, followed by CXC chemokines and hormones.

For the ≥0.7 loading values in PreVPo (Figure 5), CC chemokines and hematopoietic markers grouped together correlating with factor 1, along with some proinflammatory cytokines. Factor 2 is dominated by CXC chemokines and a hematopoietic marker. For factor 3, the only markers that remain above the strong ≥0.7 loading value are the hormones.

### Factors for post-stress vs. recovery in acute stress response

For the PoVRe stage of acute stress, ≥0.3 and ≥0.7 loadings revealed four factors (Figures 3, 6). In our ≥0.3 loading analysis (Figure 3), there were noted potent proinflammatory markers and cellular activation (IL-2, IL-6, TNF-beta, CD40L, TNF-alpha, and IFN-gamma), CC chemokines (Eotaxin, RANTES, MCP3), CXC chemokines (Gro-alpha, IP10, Fractalkine) and hematopoietic markers (GMCSF, IL-5, Flt3L). Additionally, cytokines associated with the Th2 response also in factor 1 (IL-4, IL-9). For factor 2, additional proinflammatory markers were present. Similar to the PreVPo phase, in factor 3, we note pro-inflammatory cytokines along with our grouping of hormones and a positive loading for the cytokine fractalkine. Factor 4 resulted in markers with very mixed functions from CC/CXC chemokines with a strong skewing toward macrophage activation and proliferation markers, to hematopoietic markers to the hormone progesterone. Worth noting for our ≥0.3 loading analysis (Figure 3), the change in fractalkine for this stage of the acute stress response is correlated with all factors. Fractalkine is a transmembrane chemokine that is produced by microglia in the CNS and has a function to chemo-attract peripheral blood mononuclear cells and is involved in tissue healing following injury (26). It has been found to regulate neuronal and glial responses to oxidative stress (27) and also has been implicated as a stress resilience molecule associated with lower predisposition to PTSD (28).

When the factor loading threshold is ≥0.7 for PoVRe (Figure 6), we see that the change in the same proinflammatory cytokines correlated in our PreVPo factor 1 along with additional hematopoietic markers. For factor 2, the only immune marker resulting was IL-1-β. IL-1-β is a known proinflammatory cytokine that is involved in tissue repair and neuronal differentiation (29). This could be playing a role in acute stress recovery. Factor 3 shows us T3, T4, Testosterone and estradiol grouping together in this acute stress response phase. For factor 4, we have MIP1b, which can induce the release of proinflammatory cytokines and has been shown to recruit CD4+ T cells.

### Factors for pre-stress vs. recovery in acute stress response

For both ≥0.3 and ≥0.7 loading values applied to our PreVRe data sets, three factors resulted (Figures 4, 7). In both loading value analyses, biomarkers with negative correlations were noted in factor 1, but were only seen in factor 3 for the ≥0.3 threshold. Positive loadings for both loading values consisted of a mix of CXC chemokines (Gro alpha, IP10, Fractalkine), peripheral hematopoietic markers (VEGFA, PDGFAA) and proinflammatory cytokines (IL-6, IL-18, etc). Negative loadings were associated with some CC chemokines, hematopoietic markers and those associated with adaptive immunity (sCD40L) or eosinophilic responses (IL-5). Negative loadings for hormones were no longer present in the ≥0.7 group. Factor 3 contained mostly hormones and CXC chemokines in our ≥0.3 analysis (Figure 4C), but only consisted of hormones once the loading value was increased to ≥0.7 (Figure 7C). Since the PreVRe principal components analysis is on a different time scale, of immediately before stress to 1 h after stress, it is more challenging to interpret these results. Altogether, the results suggest that the shift in these biomarkers from pre-stress to recovery is determined by a proinflammatory, CXC chemokine response that involves some markers associated with hematopoiesis, followed by hormones. The data suggests that some of the CC chemokines and adaptive immunity (Th2) markers present here may work toward a more Th2 vs. a Th1 response to the group of biomarkers that are positively affected during the proinflammatory acute stress response event.

### Summary

Our results showed a general trend in all phases analyzed (PrevPo, PovRe, PrevRe) for CC chemokines in factor 1, followed by CXC chemokines in factor 2 and hormones either in factor 2 or factor 3,4 (Figures 2–7). The CXC group, which contains interleukin IL-8, predominantly attracts neutrophils whereas the CC group, which contains chemokines regulated upon activation normal T cell expressed and secreted (RANTES) and monocyte chemoattractant protein 1 (MCP1), predominantly attracts monocytes and lymphocytes (30). Our results suggest that in the acute stress response, markers for monoctye and lymphocyte attraction are present in factor 1 for both PreVPo and PoVRe phases. Since adaptive immunity markers are seen in factor 1 for PreVPo and PoVRe phases (Figures 2A, 3A, 5A, 7A), it is possible that if acute stress is able to stimulate a stress inoculation event (31), that a good majority of these participants already have markers primed for adaptive response to stress. Therefore both monoctye and lymphocyte recruitment and neutrophil recruitment occur during the acute stress response. We also see a consistent trend of hormones grouping together in their own factor, representing the HPA/HPG axes' importance in the acute stress response.

Worth noting is that while some stress studies have detected quick increases in pro-inflammatory cytokines in the plasma (32), identification of rapid cytokine changes in the saliva in acute stress is relatively novel. Immune cells in the oral mucosa respond to wounds or infection more quickly than in other tissues such as the skin. Within minutes of an oral mucosa wound, the inflammatory process begins leading to the release of chemokines, pro-inflammatory cytokines, and cellular infiltration (33). Oral mucosal mast cells contain some pre-formed cytokines, such as TNF-alpha, in their granules (34). Mast cell degranulation in brain and heart tissue has been shown to occur under acute stress (35). One could conceive that mast cells, oral mucosa, and other immune cells become activated under acute stress, releasing preformed cytokines contained in granules. This activation would lead to the transcription of other cytokines and chemokines by resident immune cells. The timing of cytokine/chemokine production after transcription initiation could vary depending on the strength of the activation, the subject, and the tissue.

### Limitations

This study is not without limitations. Our relatively small sample size of 63 participants serves as a pilot study for this investigation and should be expanded to a larger group, including more diverse age and ethnic brackets. We also had a population consisting mostly of male subjects, so increasing the number of females in the study is advisable. Another consideration is the fact that this population consists of all individuals with prior EMS or military experience, not reflective of the general population. Saliva sampling, even though it is less invasive and costly than drawing blood or tracing protein levels in the brain, does have its draw backs as it may not be reflective of all of the signals initiated following acute stress. Additionally, this is a very unique type of stress that is being tested, involving aspects of both physical and emotional stress, so this acute stress response may not apply to all types of stress. Additionally, we did not investigate all immune markers that may be involved in the acute stress response. Furthermore, we only investigated a representative panel of immune markers, rather than the full set of signals produced by the immune system. Also, since we collected salivary samples within a narrow window of time (immediately before stress to 1 h after stress test) we may not have captured the nuances in immune marker shifts due to the fact that some immune cell mediators are short lived, degrade quickly or are difficult to detect in assays (36). We also acknowledge that both non-standardization of flow rate, along with stimulated saliva collection method could introduce potential error through changes in pH. This could be investigated in future experiments.

### Future directions

Future directions should focus on characterizing each factor based on the combination of immune mediators that are present. Identifying the molecular mechanistic roles that each of the factors play in the acute stress response could yield insight into methods for detecting individuals who are vulnerable to stress, or discover potential actionable targets for treating stress related disorders and stress related breakdowns.

## Data availability statement

The original contributions presented in the study are included in the article/Supplementary materials, further inquiries can be directed to the corresponding author.

## Ethics statement

The studies involving human participants were reviewed and approved by Rocky Vista University IRB Committee. The patients/participants provided their written informed consent to participate in this study.

## Author contributions

RR, CW, EO, and FT wrote and edited the manuscript. RR and AL conceptualized experimental design. RR performed experiments. MP performed statistical analyses. All authors contributed to the article and approved the submitted version.

## Funding

This grant was funded through an awarded intramural grant from Rocky Vista University.

## Conflict of interest

The authors declare that the research was conducted in the absence of any commercial or financial relationships that could be construed as a potential conflict of interest.

## Publisher's note

All claims expressed in this article are solely those of the authors and do not necessarily represent those of their affiliated organizations, or those of the publisher, the editors and the reviewers. Any product that may be evaluated in this article, or claim that may be made by its manufacturer, is not guaranteed or endorsed by the publisher.
