# Supplementary material for: Principal component analysis of salivary cytokines and hormones in the acute stress response
Source: Front Psychiatry. 2022 Oct 19;13:957545. doi: 10.3389/fpsyt.2022.957545 (PMC9626986; doi:10.3389/fpsyt.2022.957545)
Supplement: Supplementary file 1 [file Data_Sheet_1.docx]

**Supplemental 1.** Assay sensitivity (minimum detectable concentrations, pg/mL) of Eve Technologies’ HD42 assay. Minimum Detectable Concentration (MinDC) is calculated using MILLIPLEX® Analyst 5.1. It measures the true limits of detection for an assay by mathematically determining what the empirical MinDC would be if an infinite number of standard concentrations were run for the assay under the same conditions.

| **Cytokine** | **MinDC (pg/mL)** | **MinDC + 2SD (pg/mL)** |
| --- | --- | --- |
| EGF | 2.8 | 4.6 |
| FGF-2 | 7.6 | 11.8 |
| Eotaxin | 4.0 | 6.8 |
| TGFα | 0.8 | 1.2 |
| G-CSF | 1.8 | 3.3 |
| Flt-3L | 5.4 | 7.0 |
| GM-CSF | 7.5 | 15.0 |
| Fractalkine | 22.7 | 37.7 |
| IFNα2 | 2.9 | 4.8 |
| IFNy | 0.8 | 1.1 |
| GRO | 9.9 | 14.1 |
| IL-10 | 1.1 | 1.6 |
| MCP-3 | 3.8 | 6.4 |
| IL-12P40 | 7.4 | 12.7 |
| MDC | 3.6 | 7.1 |
| IL-12P70 | 0.6 | 1.0 |
| IL-13 | 1.3 | 1.9 |
| IL-15 | 1.2 | 1.7 |
| sCD40L | 5.1 | 9.9 |
| IL-17 | 0.7 | 1.2 |
| IL-1RA | 8.3 | 17.1 |
| IL-1α | 9.4 | 12.6 |
| IL-9 | 1.2 | 2.0 |
| IL-1β | 0.8 | 1.0 |
| IL-2 | 1.0 | 1.6 |
| IL-3 | 0.7 | 1.0 |
| IL-4 | 4.5 | 7.1 |
| IL-5 | 0.5 | 0.7 |
| IL-6 | 0.9 | 1.3 |
| IL-7 | 1.4 | 2.4 |
| IL-8 | 0.4 | 0.7 |
| IP-10 | 8.6 | 14.0 |
| MCP-1 | 1.9 | 3.4 |
| MIP-1α | 2.9 | 6.2 |
| MIP-1β | 3.0 | 4.8 |
| TNFα | 0.7 | 1.1 |
| TNFβ | 1.5 | 1.9 |
| VEGF | 26.3 | 47.9 |
| PDGF-AA | 0.4 | 0.7 |
| PDGFAB-BB | 2.2 | 2.7 |
| RANTES | 1.2 | 1.9 |

**Supplemental 2.** Assay sensitivity (minimum detectable concentrations, pg/mL) of Eve Technologies’ STTHD assay. Minimum Detectable Concentration (MinDC) is calculated using MILLIPLEX® Analyst 5.1. It measures the true limits of detection for an assay by mathematically determining what the empirical MinDC would be if an infinite number of standard concentrations were run for the assay under the same conditions.

| **Analyte** | **MinDC (ng/mL)** | **MinDC + 2SD (ng/mL)** |
| --- | --- | --- |
| Cortisol | 0.17 | 0.33 |
| Estradiol | 0.01 | 0.01 |
| Progesterone | 0.14 | 0.25 |
| Testosterone | 0.08 | 0.15 |
| T3 | 0.08 | 0.12 |
| T4 | 0.24 | 0.51 |

**Supplemental 3.** Descriptive statistics data for each analyte measured and each time point (T1=pre, T2=post, T3=recovery).

------------------------------------------- Time=1 --------------------------------------------

The MEANS Procedure

Variable Mean Std Dev N Median Minimum Maximum

---------------------------------------------------------------------------------------------

EGF 355.2221053 164.1357052 57 310.4300000 122.4100000 827.1100000

FGF2 53.0878947 31.2399031 57 46.2500000 0 193.1500000

Eotaxin 3.3138596 2.2239907 57 3.0000000 0.2400000 17.0400000

TGFa 1.3464912 0.9642123 57 1.1300000 0 4.7200000

GCSF 13.7212281 14.6334232 57 8.9700000 0 79.6400000

Flt3L 3.4666667 2.0542372 57 3.3200000 0 9.1400000

GMCSF 1.4480702 1.2905417 57 1.2800000 0.2400000 9.7300000

Fractalkine 99.5461404 56.3060598 57 87.6000000 17.0600000 278.4400000

IFNa2 7.5010526 12.9523763 57 4.5000000 0 93.1100000

IFNy 1.1587500 1.9111577 56 0.5250000 0 8.1200000

GROalpha 366.8505263 269.5950795 57 294.4200000 41.4200000 1508.06

IL10 1.4368421 1.3322352 57 1.0100000 0.2400000 7.1300000

MCP3 6.5714035 5.7530829 57 6.2000000 0 24.7200000

IL12P40 2.0943860 4.7450184 57 0.6400000 0 33.5700000

MDC 74.8182456 43.4724738 57 83.7100000 6.5000000 212.0400000

IL12P70 0.8531579 0.9717347 57 0.8400000 0 6.4900000

PDGFAA 50.2043860 35.5987766 57 43.2900000 3.3800000 166.9200000

IL13 0.5198246 0.6108352 57 0.3600000 0 2.1100000

PDGFBB 1.2210526 2.2975777 57 0.7800000 0 13.0900000

IL15 1.0556140 0.6001131 57 0.9500000 0.2100000 4.3300000

sCD40L 0.7391228 0.7780385 57 0.5700000 0 5.1700000

IL17A 0.0712281 0.3713781 57 0 0 2.7500000

IL1RA 5329.39 3178.75 57 4349.98 1548.12 13207.75

IL1a 186.1110526 148.7472679 57 143.0300000 12.2600000 543.9600000

IL9 0.1210526 0.1497341 57 0.1200000 0 0.9600000

IL1B 7.0833333 8.4060232 57 3.6000000 0.6000000 35.6000000

IL2 0.1707018 0.2326698 57 0.1300000 0 1.6500000

IL3 0.0029825 0.0225171 57 0 0 0.1700000

IL4 11.4235088 9.1600668 57 10.8500000 1.5500000 62.8000000

IL5 0.1577193 0.0980164 57 0.1500000 0.0300000 0.6200000

IL6 0.6650877 0.7276703 57 0.4100000 0 3.6100000

IL7 1.0945614 0.5959808 57 0.9000000 0.2600000 2.3400000

IL8 129.5989474 180.2061529 57 64.8500000 8.2400000 1048.98

IP10 633.4556140 552.9066011 57 426.9200000 94.8100000 2742.03

MCP1 616.9275439 1495.40 57 225.6200000 91.6800000 9071.60

MIP1a 1.0663158 1.5768862 57 0 0 6.4500000

MIP1B 0.6529825 1.7970422 57 0 0 12.2200000

RANTES 3.3252632 1.8522131 57 3.0300000 0 9.2900000

TNFa 2.3905263 2.2713207 57 1.6600000 0.3000000 9.5800000

TNFB 0.7078947 0.9067283 57 0.4000000 0 5.2400000

VEGFA 96.9401754 47.5493163 57 92.2900000 23.7100000 200.1400000

IL18 102.2054386 195.3880550 57 40.6800000 0 1373.65

Cortisol 10.1685965 7.6020658 57 8.6200000 0 35.0900000

Estradiol 0.4633333 0.2388988 57 0.4200000 0.0500000 1.3000000

Progesterone 11.4133333 7.3191667 57 9.8800000 0.0300000 33.5400000

T3 1.5643860 1.2619529 57 1.2900000 0 7.6300000

T4 6.0673684 4.5701092 57 5.0000000 0 20.5000000

Testosterone 1.2615789 1.0236992 57 1.0000000 0.1600000 6.0600000

---------------------------------------------------------------------------------------------

------------------------------------------- Time=2 --------------------------------------------

Variable Mean Std Dev N Median Minimum Maximum

---------------------------------------------------------------------------------------------

EGF 450.8720339 192.5877556 59 411.0000000 227.3500000 1319.39

FGF2 64.4403390 30.7186148 59 58.2600000 20.6800000 156.6200000

Eotaxin 4.4867797 3.2103193 59 3.6000000 0.6400000 18.3400000

TGFa 1.9396610 1.1105621 59 1.7000000 0.1500000 5.0800000

GCSF 17.1149153 14.9877128 59 12.8600000 2.3300000 86.9200000

Flt3L 3.8006780 2.2262469 59 3.5000000 0 9.2600000

GMCSF 1.9262712 1.7577208 59 1.6000000 0.1900000 10.9900000

Fractalkine 204.4022034 153.3050467 59 163.3500000 33.9400000 878.0800000

IFNa2 6.3855932 4.6135607 59 5.4700000 0 23.9000000

IFNy 1.4040678 2.3264040 59 0.5500000 0 11.0100000

GROalpha 671.3030508 432.1211123 59 556.9600000 102.4900000 2477.50

IL10 2.8038983 2.1956525 59 2.1200000 0.0800000 10.9800000

MCP3 8.9947458 5.7599914 59 8.7100000 0 26.5400000

IL12P40 3.2262712 6.8279857 59 1.2600000 0 36.7900000

MDC 82.1742373 42.8999957 59 89.6000000 13.2800000 196.5600000

IL12P70 1.1906780 1.3055873 59 0.7700000 0 7.4100000

PDGFAA 118.2457627 84.7147318 59 94.9900000 13.8700000 408.6500000

IL13 0.5530508 0.6088181 59 0.4700000 0 2.3900000

PDGFBB 1.5805085 2.3933388 59 0.9400000 0 13.1500000

IL15 1.7471186 1.0232454 59 1.3700000 0.6400000 6.1600000

sCD40L 1.1900000 1.0965872 59 0.8900000 0 5.9900000

IL17A 0.2011864 0.6600237 59 0 0 3.8200000

IL1RA 5229.38 2434.96 59 4655.24 1835.58 13484.62

IL1a 238.8042373 153.0305058 59 215.7100000 35.1900000 700.8000000

IL9 0.1594915 0.2005374 59 0.1400000 0 1.0300000

IL1B 10.6105172 10.7219464 58 7.0050000 1.3100000 49.7400000

IL2 0.2486441 0.3155425 59 0.1600000 0 1.8200000

IL3 0.0064407 0.0354655 59 0 0 0.2300000

IL4 17.1054237 13.3241589 59 15.4100000 1.7800000 78.5000000

IL5 0.1925424 0.1232966 59 0.1700000 0.0200000 0.7500000

IL6 1.1981356 0.9669459 59 0.9700000 0 4.2600000

IL7 2.2623729 1.8658356 59 1.7600000 0.5200000 11.4600000

IL8 254.8638983 226.5757379 59 183.1900000 29.9700000 1192.66

IP10 1010.73 733.1623195 59 778.9500000 235.5100000 3902.86

MCP1 1476.80 2496.82 59 461.5200000 87.0900000 9071.60

MIP1a 2.3652542 2.4413439 59 1.9700000 0 12.2600000

MIP1B 1.8796610 3.3341028 59 0.7100000 0 16.2400000

RANTES 3.6796610 1.7738366 59 3.2800000 0.2600000 9.0300000

TNFa 3.9586441 3.5179654 59 2.6800000 0.5400000 15.3400000

TNFB 0.9940678 1.0190363 59 0.7100000 0 5.1500000

VEGFA 150.2581356 58.2703813 59 148.2100000 47.6200000 364.4300000

IL18 152.6606780 155.5272475 59 112.6400000 0 748.2000000

Cortisol 8.0019298 7.1655277 57 7.3400000 0 26.3100000

Estradiol 0.4883051 0.3289909 59 0.4300000 0.0300000 1.3200000

Progesterone 10.6152542 15.7909280 59 7.8900000 0.0200000 107.8900000

T3 2.0193220 2.1936207 59 1.5600000 0 9.2500000

T4 8.7105085 9.4778929 59 5.5300000 0 57.6600000

Testosterone 1.6408475 1.6950539 59 1.2900000 0.1400000 7.8600000

---------------------------------------------------------------------------------------------

------------------------------------------- Time=3 --------------------------------------------

Variable Mean Std Dev N Median Minimum Maximum

---------------------------------------------------------------------------------------------

EGF 288.7976271 112.9888701 59 277.6500000 87.6000000 575.3900000

FGF2 55.1645763 30.3803826 59 48.9700000 0 177.2800000

Eotaxin 4.4610345 3.5612144 58 3.6000000 0.6400000 19.1900000

TGFa 1.3825424 1.3269846 59 0.9900000 0.1000000 6.9500000

GCSF 15.8830508 15.3254284 59 11.2400000 0 67.9200000

Flt3L 3.9675862 2.4788815 58 3.5300000 0.3300000 11.8100000

GMCSF 2.1915517 2.8410134 58 1.5600000 0.1900000 15.2200000

Fractalkine 124.4128814 115.7164873 59 80.0000000 27.8500000 615.5500000

IFNa2 7.0715517 6.3685581 58 6.0400000 0 32.4100000

IFNy 2.0715254 4.6372665 59 0.6400000 0 25.0600000

GROalpha 297.8954237 230.3860881 59 255.8800000 34.9900000 1471.95

IL10 1.7320339 2.4492876 59 1.0900000 0.2400000 16.4800000

MCP3 9.3505172 7.0331120 58 7.9000000 0 31.6400000

IL12P40 4.4131034 10.5219962 58 1.2300000 0 56.0900000

MDC 94.6179661 71.2575054 59 87.5900000 13.2800000 480.4800000

IL12P70 1.4829310 2.0048835 58 0.9400000 0 10.3700000

PDGFAA 43.0101695 41.1421369 59 33.6800000 1.1200000 266.1100000

IL13 0.7606897 0.8608622 58 0.6900000 0 3.3900000

PDGFBB 2.2706897 3.3249191 58 1.5050000 0 16.8600000

IL15 1.4162069 1.2797105 58 1.1500000 0.0200000 6.8600000

sCD40L 1.2189655 1.6800863 58 0.7300000 0 8.7800000

IL17A 0.2948276 1.0669126 58 0 0 5.3100000

IL1RA 4816.43 2464.02 59 4322.34 730.4000000 10646.60

IL1a 169.9240678 137.4556987 59 134.3300000 6.7400000 590.5900000

IL9 0.1898276 0.2966804 58 0.1450000 0 1.5000000

IL1B 4.8911864 5.1656255 59 2.6800000 0.3600000 22.3500000

IL2 0.2882759 0.5023179 58 0.1450000 0 2.5200000

IL3 0.0222414 0.0984626 58 0 0 0.5200000

IL4 15.0058621 20.1414693 58 10.2950000 0.6400000 112.6300000

IL5 0.2013793 0.1916361 58 0.1650000 0.0500000 1.1000000

IL6 0.7166102 0.8845038 59 0.4300000 0 4.6600000

IL7 1.2891525 1.0437107 59 1.0100000 0.1200000 6.0900000

IL8 109.2367797 92.0190887 59 87.9100000 5.2700000 505.5900000

IP10 506.4420339 484.5981923 59 415.5100000 29.1300000 3169.01

MCP1 472.5596610 1210.69 59 203.0600000 51.0800000 9071.60

MIP1a 1.9547458 3.0895242 59 1.2000000 0 19.7400000

MIP1B 0.5491525 1.2180854 59 0 0 6.0600000

RANTES 3.9189655 2.1034264 58 3.5100000 0.9000000 11.6800000

TNFa 2.6918644 2.6066318 59 1.7300000 0.3000000 12.7900000

TNFB 1.2089655 1.6399312 58 0.7500000 0 8.4300000

VEGFA 76.5940678 53.9875043 59 64.7700000 13.9800000 305.3400000

IL18 77.4223729 126.3317301 59 32.7000000 0 622.0100000

Cortisol 9.6310169 8.6052513 59 7.3600000 0 50.0700000

Estradiol 0.4166102 0.2072447 59 0.3900000 0.0600000 1.1600000

Progesterone 11.6028814 8.1144998 59 10.4800000 0.0100000 38.2800000

T3 1.5928814 1.3802599 59 1.2900000 0 7.1700000

T4 6.2972881 4.6999313 59 5.1300000 0 20.0300000

Testosterone 1.3464407 1.3172840 59 1.0400000 0.1800000 7.5100000

---------------------------------------------------------------------------------------------
